# Pyroptosis, ferroptosis, and autophagy cross-talk in glioblastoma opens up new avenues for glioblastoma treatment

**DOI:** 10.1186/s12964-023-01108-1

**Published:** 2023-05-19

**Authors:** Sicheng Wan, Guanghui Zhang, Ruochen Liu, Muhammad Nadeem Abbas, Hongjuan Cui

**Affiliations:** 1 State Key Laboratory of Resource Insects, Medical Research Institute, Chongqing, 400715 China; 2 Chongqing Engineering and Technology Research Center for Silk Biomaterials and Regenerative Medicine, Chongqing, 400715 China; 3 Engineering Research Center for Cancer Biomedical and Translational Medicine, Southwest University, Chongqing, 400715 China; 4 Jinfeng Laboratory, Chongqing, 401329 China

**Keywords:** Glioblastoma (GBM), Pyroptosis, Ferroptosis, Autophagy, Molecular mechanism, Temozolomide (TMZ), Drug tolerance

## Abstract

Glioma is a common primary tumor of the central nervous system (CNS), with glioblastoma multiforme (GBM) being the most malignant, aggressive, and drug resistant. Most drugs are designed to induce cancer cell death, either directly or indirectly, but malignant tumor cells can always evade death and continue to proliferate, resulting in a poor prognosis for patients. This reflects our limited understanding of the complex regulatory network that cancer cells utilize to avoid death. In addition to classical apoptosis, pyroptosis, ferroptosis, and autophagy are recognized as key cell death modalities that play significant roles in tumor progression. Various inducers or inhibitors have been discovered to target the related molecules in these pathways, and some of them have already been translated into clinical treatment. In this review, we summarized recent advances in the molecular mechanisms of inducing or inhibiting pyroptosis, ferroptosis, or autophagy in GBM, which are important for treatment or drug tolerance. We also discussed their links with apoptosis to better understand the mutual regulatory network among different cell death processes.

Video Abstract

**Supplementary Information:**

The online version contains supplementary material available at 10.1186/s12964-023-01108-1.

## Background

Glioblastoma multiforme (GBM), a World Health Organization (WHO) grade IV glioma, is the most common and devastating primary central nervous system (CNS) malignant tumor. In 2020, there are around 300,000 newly diagnosed cases of brain tumors and nervous system cancers worldwide, with roughly 250,000 deaths [1]. GBM accounts for 48% of all CNS tumors. According to statistical data from 2015 to 2020, the annual average incidence of GBM in the USA was 3.21 cases per 100,000 people [2]. Several statistical reports from the National Cancer Institute (NCI) and the Centers for Disease Control and Prevention (CDC) have also shown that the 65- to 85-year-old age group has the highest incidence of GBM among adults and elderly populations [3]. According to additional statistics, the incidence of GBM is highly correlated with gender, with an annual age-adjusted incidence of only 2.53 for women versus 4 for men per 100,000 people [4]. Although a number of GBM treatments, including immunotherapy, targeted precision therapy, and supportive therapy, have improved the short-term survival rate of GBM patients to some extents, the overall patient prognosis remains poor, particularly the five-year survival rate. In addition, along with the deterioration from the disease, the function of the nervous system is gradually destroyed, and the resulting complications will have a devastating impact on the quality of patients' life and their families [5].

The main factors contributing to the unfavorable prognosis include advanced age, poor physical fitness, and the limitations in surgical resection of GBM lesion sites. The progression-free survival (PFS) after combining chemotherapy is less than 6 months in older patients [6]. Furthermore, because of the complex heterogeneity of GBM, and unique characteristics of the blood–brain barrier (BBB) and blood–brain tumor barrier (BBTB) structures in the brain, most chemotherapeutic agents cannot smoothly reach the lesion and accumulate to a sufficient concentration in the tumor microenvironment (TME), severely limiting GBM treatment [7]. The current standard clinical treatment for GBM is maximum surgical resection followed by chemotherapy. Such a treatment scheme can increase the median survival time (MST) of GBM patients from 4 to 15 months, while  keeping the 5-year survival rate at 5%. Although novel drugs such as CAR-T, CAR-NK [8], and oncolytic viruses [9], among others, are gradually investigated in GBM clinical trials, temozolomide (TMZ) remains the major option for clinical chemotherapy. Numerous studies have demonstrated that long-term TMZ treatment results in strong drug tolerance, which is related to the methylation of O6-methylguanine-DNA methyltransferase (MGMT), the isocitrate dehydrogenase (IDH) mutation, the 1p/19q co-deletion status, as well as many abnormal signaling cascades [10, 11].

Initially, tumorigenesis was thought to be caused by the overactivation of proto-oncogenes or the inactivation of tumor suppressor genes, which disrupt normal cell proliferation and differentiation. Researchers have found that tumorigenesis is linked not only to abnormal proliferation, but also to the blockage or defect in cell death process. The latest cell death classification was modified in 2018 by the Nomenclature Committee on Cell Death (NCCD), which split cell death into accidental cell death (ACD) and regulated cell death (RCD) based on differences in cell death regulatory manner [12]. Programmed cell death (PCD) is a kind of RCD that can be activated by external factors such as pathogens or drugs and is tightly regulated by a number of intracellular molecules and pathways [13]. Pyroptosis, ferroptosis, and autophagy are three newly discovered types of PCD that are being studied in cell models for many diseases. Their activation or inhibition is closely related to the occurrence of all cancers, including GBM. Combining inhibitors or activators of pyroptosis, ferroptosis, and autophagy (which balance the abnormal dysregulations of these processes) with TMZ may reduce drug tolerance and produce a better therapeutic impact, a number of similar studies are also under way in clinical trials. In the following chapters, we expounded on the molecular mechanisms of pyroptosis, ferroptosis, and autophagy, reviewed their studies in GBM over the last two years, and discussed how to best exploit these pathways to synergistically improve the effect of traditional or novel GBM treatment strategies.

## Pyroptosis and Glioblastoma

Pyroptosis is a new form of PCD that is thought to be involved in the body’s defense against pathogens. In contrast to immunosuppressed apoptosis, pyroptosis is characterized by cell expansion until membrane rupture, which results in the release of cytokines and the activation of a cascade of inflammatory and immune responses. The key morphological features of pyroptosis are cellular swelling, vesicular bulge formation, membrane perforation, and final loss in cell integrity. Pyroptosis can be executed by a number of members of the Gasdermin family and is classified into classical and nonclassical pyroptosis types based on whether caspase-1 is activated along the pathway [14, 15].

### Inflammasome and the Activation of Caspase-1

The inflammasome, a multiple-protein complex, is an important component of the natural immune system. Previous research has reported five types of inflammasomes: NLRP1, NLRP3, NLRP4, IRAF, and AIM2, all of which share the adaptor, the effector caspase, and the NOD-like receptor (NLR) family protein (NLRP1/3/4) as the receptor. It has also been shown that NLRP3 plays a pivotal role in the body’s immune response and the development of immune-type disease since it can be activated by multiple pathogens [16, 17]. Pattern recognition receptors (PRRs) of the inflammasome play a vital role in pyroptosis by recognizing pathogen-associated molecular patterns (PAMPs), which are highly conserved molecular structures on the surface of pathogenic microorganisms such as lipopolysaccharides, peptidoglycans, and teichoic-acids, as well as other damage-associated molecular patterns (DAMPs) [18, 19]. Then, the Toll-like receptors (TLRs), such as TLR4, are activated to activate interleukin-1 receptor associated kinase (IRAK-1) and transforming growth factor-β-activated kinase 1 (TAK1) by myeloid differentiation factor 88 (MyD88), which triggers the NF-kB kinase inhibitor (IKK) complex to phosphorylate the inhibitor of NF-kB (IkB) and induce its degradation via the ubiquitin- proteasome pathway. Finally, the c-REL–p52 or RELA–p50 NF-kB complex is released to the nucleus to activate the transcription of their target genes, such as the inactive interleukin precursors pro-IL-1β, pro-IL-18, and pro-caspase-1. Meanwhile, pro-caspase-1 will be recruited and activated by the ASC domain of the inflammasome, cleaves itself into active caspase-1, which is involved in the maturation of interleukin precursor molecules, resulting in a widespread inflammatory response [17, 20].

### Gasdermins- the Pyroptosis Executors

Humans have six members of the Gasdermin family: Gasdermin (A-D), Gasdermin E (also known as the DFNA5), and DFNB59 [21]. They all have two conserved domains, the N-terminus action domain and the C-terminus functional inhibition domain. The N-terminus, as a primary functional domain, is engaged in the pyroptosis process, while the C-terminus contains the autoinhibitory. Under normal physiological conditions, the C- and N-termini maintain the interactional state to inhibit the cell membrane perforation function of the N-terminal domain. After being stimulated by external signals, gasdermins (A-E) will be cleaved by caspase-1/4/5/11, resulting in the dissociation of the N-terminal domain. The N-terminal domain targets the cell membrane and causes membrane perforation by binding to phosphatidylinositol, phosphatidic acid, and phosphatidylserine. The rupture of the cell membrane causes changes in the osmotic pressure inside and outside of the cell, resulting in a massive outflow of potassium ions and membrane potential instability. Eventually, a large number of pro-inflammatory cytokines, such as IL-1β and IL-18, were released into the extracellular space, producing an intense inflammatory response. In the nonclassical pyroptosis pathway, inflammatory factors in the cytoplasm, such as TLR, can directly activate caspase-4/5/11 (caspase-11 in mice) to induce the cleavage of GSDMD and expose its N-terminal domain, so initiating the pyroptosis process. This also reveals that caspase-4/5/ and 11 play a critical role in the host’s defense against gram-negative bacteria. Pannexin-1 has been found to participate in the nonclassical pyroptosis pathway through the purinergic receptor P2X7 to regulate iron flow [22–25]. However, according to the investigations, other caspase family members other than caspase-1, do not have the function of transforming IL-1β and IL-18 to maturity (their maturity determines the level of immunoreaction).

### Pyroptosis, a Novel GBM Treatment Strategy

Although the basic research on pyropotosis in GBM treatment is limited, the published studies have shown that certain genes (miRNAs and ncRNAs), their coding production, and drugs can trigger pyropotosis in GBM in vitro.

Several recent studies have found that specific noncoding RNAs (ncRNAs) regulate multiple molecules in the pyroptosis pathway. Long noncoding RNA (lncRNA): *hsa-circ-0001836* is upregulated in GBM, possibly indicating its relationship with carcinogenesis. A recent study demonstrated that the knockdown of *hsa-circ-0001836* reduces GBM cell proliferation and at the same time increases *NLRP1* expression by demethylating its promoter region, up-regulating caspase-1 and the inflammatory molecules IL-1B and IL-18 [26]. Moreover, *miRNA-214* has been linked to pyroptosis in GBM through directly decrease caspase-1 mRNA stability and translation. Transfecting its inhibitor into GBM cells suppresses cell proliferation and migration in a caspase-1 dependent manner [27]. Furthermore, several studies have shown that certain drugs and small molecular inhibitors can induce pyroptosis in GBM. The GBM cell lines LN-229, U87-MG, and U251 after treatment with galangin [28], kaempferol [29], benzimidazoles [30], 4,5-Dimethoxycanthin-6-one [31] (new LSD1 inhibitor), and AT7519 [32] (multi-CDKs inhibitors) can induce obvious pyroptosis, and mouse cell-derived xenograft (CDX) model experiments have also illustrated their anti-tumor role. J.Y. et al. developed a novel controllable drug carrier named TMZ magnetic temperature-sensitive liposomes (TMZ/Fe-TSL). When GBM is subjected to an alternating magnetic field (AMF), caspase-1, GSDMD, and NLRP3 will be activated, resulting in pyroptosis [33].

Furthermore, the conventional viewpoint holds that the kill cells containing numerous cytotoxic granules that can remove tumors by inducing cell apoptosis. A recent study pointed out that the inclusions released by kill cells can cause pyroptosis by activating gasdermin-E (GSDME). These cells have high levels of perforin and Granzyme B (Gzm B), and with the help of perforin, Gzm B can be delivered into cancer cells directly or indirectly, via coordinating with caspase-3, cleave the identical amino acid site D270 of the N-terminal domain of GSDME, activating pyroptosis. Then, a series of anti-tumor immune responses are stimulated, which inhibit tumor growth. Caspase-3 can also be stimulated by tumor necrosis factor α (TNF- α) or cancer chemotherapeutic drugs to induce apoptosis [34, 35]. Hence, cancer cells expressing high leveled GSDME may be more susceptible to pyroptosis. It has also been shown that the promoter region of *GSDME* is highly methylated in a variety of cancers. Inhibiting DNA methylation will upregulate the expression of *GSDME* and improve the killing potential of chemotherapy drugs against cancer cells. The Cancer Genome Atlas (TCGA) bulk RNA-seq data also showed that the mRNA expression level of *GSDME* in cancer tissues is lower than that in normal tissues (TCGA-pan cancer) [21, 36, 37]. However, when compared to normal brain tissue in the GTEx database, we surprisingly found that *GSDME* is highly expressed in GBM patients’ tissues in the TCGA-GBM cohort. Meanwhile, other pyroptosis markers such as *GSDED*, *CASP1*, *GSDMC*, and *GSDMA* also have higher expression level in GBM (Supplementary Fig. 1A), implying that inducing pyroptosis can be acted as an effective approach for GBM treatment, but also implying that pyroptosis may be related to the inhibitory tumor immune microenvironment (TIME) in GBM. According to the past researches, many immune inhibitory cells, such as regulatory T cells, M2 like macrophages, and myeloid-derived suppressor cells, infiltrate the TME of GBM. These cells interfere with immune cells’ ability to kill tumor cells and impact the immunotherapy effect [38]. There is evidence that another type of inflammatory cell death-necroptosis, may aggravate the TIME of glioma. Future studies should focus on the relationship between pyroptosis and TIME in GBM.

**Fig. 1:**
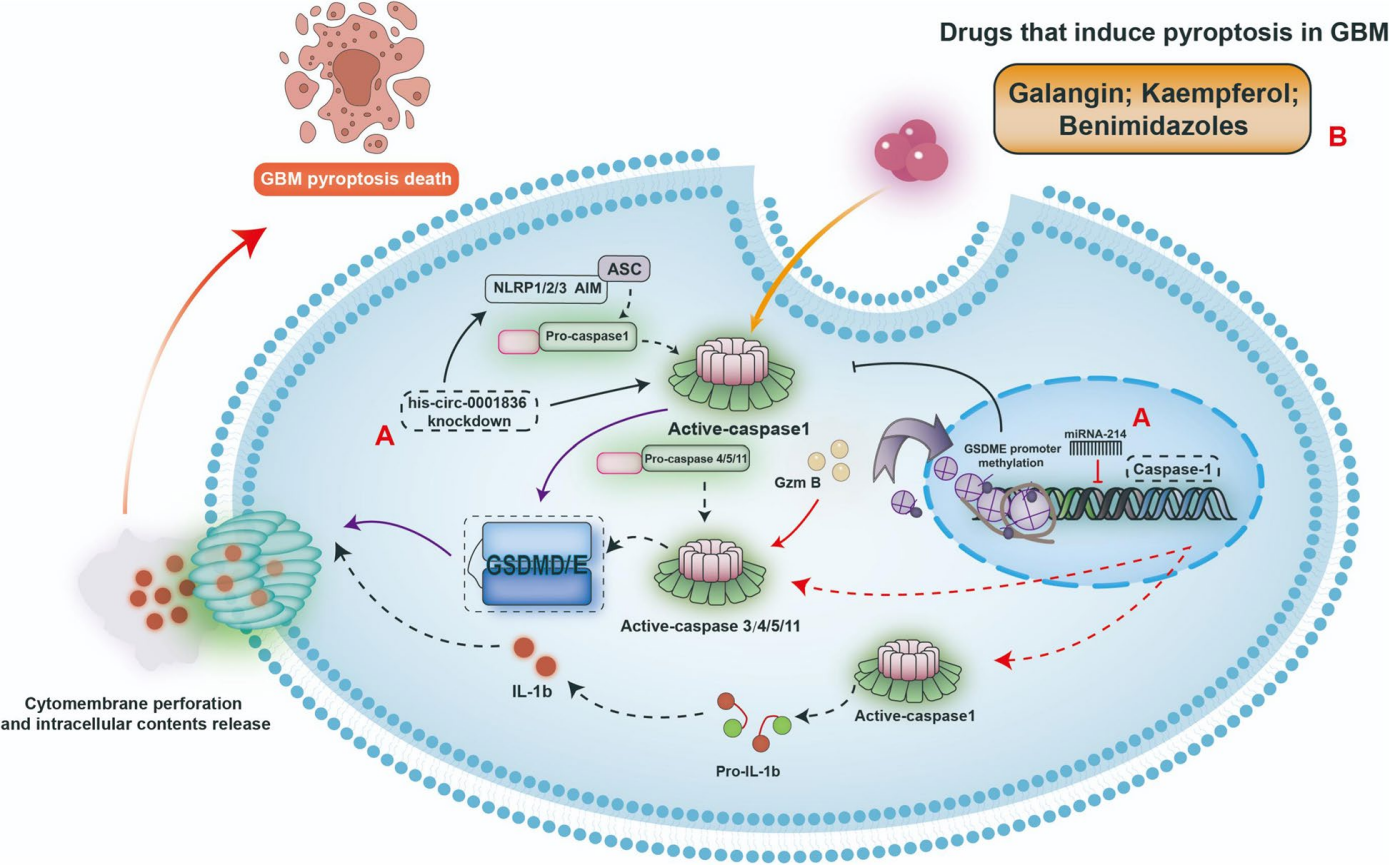
The molecular basis of pyroptosis and its regulation mode in GBM. **A** The miRNA-214 negatively regulates the mRNA of caspase-1. The Knockdown his-circ-0001836 activates caspase-1 and reduces the methylation of the NLRP1 promoter region. **B** The drugs: Galangin, Kaempferol, and Benimidazoles can induce pyroptosis in GBM by activating caspase-1

Pyroptosis, a newly discovered type of PCD, has only recently begun to be widely explored in many types of human cancer, including GBM. As shown in Fig. 1, pyroptosis is involved in a number of inflammatory reactions in GBM and can be induced by multiple drugs or inhibited by ncRNAs. The pathological detection of pyroptosis-related molecules may have important value for the prognostic diagnosis of GBM patients and the design of post-surgery treatment plan. On account of GBM is characterized by resistance to drug-induced apoptosis, research into the mechanisms and inducers of pyroptosis may contribute to the development of new GBM treatment strategies.

## Ferroptosis and Glioblastoma

Dixon discovered ferroptosis, a novel cell death program, in 2012 [39]. It is characterized by cell death and accompanied by a high iron and reactive oxygen species (ROS) dependence [40]. The fundamental mechanism of ferroptosis is that highly expressed polyunsaturated fatty acids (PUFAs) are catalyzed on the cell membrane in the presence of ferrous ions and lipoxygenase (LOX), resulting in lipid peroxidation [41]. Secondly, LOX catalyzes the formation of peroxides and fatty acids from PUFAs while also producing a large amount of ROS [42]. Ferroptosis-dependent cell death is mainly caused by oxidative damage, followed by cell membrane damage and fracture caused by ROS attack.

Cell death mediated by ferroptosis has morphological features such as cell membrane breakage, decreased mitochondrial volume, increased outer membrane density, and the disappearance of cristae. When cell undergo ferroptosis, morphological changes in the nucleus are not visible, which is a distinct form from apoptosis [43–47]. Changes in the chemical composition are mostly manifested by increased levels of lipid peroxidation and intracellular ROS [48], decreased cysteine intake, and glutathione depletion. Ferroptosis is also characterized by an evident drop in nicotinamide adenine dinucleotide phosphate (NADPH). Moreover, ferroptosis is related to a number of classical pathways, such as the ROS-MAPK [49], TP53 [50], and Hippo [51] pathways.

### Abnormal Iron Metabolism in GBM

Several pivotal star molecules are involved in the occurrence of ferroptosis in cancer cells. Iron is an important central atom that is located in the catalytic functional domain of certain enzymes, which participate in multiple cellular metabolic reactions. The growth of hypermetabolic requirement tumors, including GBM, is strongly dependent on iron as compared to normal cells [52, 53]. Transferrin (TF) binds to free irons in the extracellular space and transport them into cells. A study has found that TF is typically overexpressed in cancer cells [54]. A high dietary iron intake increases the risk of colorectal cancer and breast cancer [55]. In reverse, a large number of free divalent iron ions in cells undergo the Fenton reaction with hydrogen peroxide, producing free hydroxyl radicals and inducing ferroptosis. Pseudolaric acid B (PAB) upregulates the expression of the transferrin receptor (TFR) to increase the intracellular iron levels, inducing ferroptosis in GBM. Moreover, the increased iron level activates NADPH oxidase 4 (NOX4), resulting in the production of lipid peroxides (NOX converts NADPH to NADP + and releases electrons to generate peroxide ions, which participate in the formation of hydrogen peroxide). Furthermore, PAB consumes glutathione (GSH) via the P53-xCT pathway, which aggravates the accumulation of lipid peroxides [56]. Because divalent metal transporter 1 (DMT1) transports the majority of ferrous irons in cells into the labile iron pool (LIP), researchers believe that targeting LIP to trigger tumor ferroptosis is feasible [57]. However, specific biomarkers are needed to identify tumors with a high LIP level in order to apply effective LIP-targeted clinical therapy. Z.N. et al. developed the 18F-TRX LIP sensor transmitter, which can perfectly detect the LIP level in the U251-MG GBM cell line. In addition, the combination of 18F-TRX with a LIP inducer has a strong anti-tumor effect [58]. Accordingly, the increased iron reliance can be regarded as a promising target for treating GBM by inducing ferroptosis.

### Molecules Associated with Ferroptosis in GBM

#### Glutathione Geroxidase 4

The glutathione peroxidase (GPX) family consists of eight members, designated as GPX1-8 [59]. Within them, Glutathione peroxidase (GPX4) is the core regulator of ferroptosis. GPX4 uses GSH as its substrate to degrade small molecular peroxides and some lipid peroxides, inhibiting intracellular lipid peroxidation [60]. (1S,3R)-RSL3, also known as RSL3, is a well-known ferroptosis inducer that can directly suppress GPX4 to induce ferroptosis [61]. The mevalerate (MVA) pathway affects selenocysteine, the central amino acid of GPX4, to induce ferroptosis [62]. After utilizing disulfiram (DFS) to treat GBM cells, the expression of GPX4 was reduced, which induced the production of ROS and increased the sensitivity of GBM cells to ferroptosis [63].

#### Cystine/Glutamate transporter

GSH is an indispensable cofactor of GPX4, and its synthesis is dependent on cystine/glutamate transporters (system xc^−^, xCT) that transport cystine from the extracellular to the intracellular. Thus, xCT overexpression leads to cells with resistance to ferroptosis [64], and it has been shown that xCT is evidently upregulated in glioma patients [65]. The xCT is composed of SLC3A2 and SLC7A11 subunits, both of which are embedded in the cell membrane. Between the two subunits, SLC7A11 serves a more vital function and has received more attention. The well-known tumor suppressor gene *TP53* downregulates *SLC7A11* transcription to induce ferroptosis in cancer cells [50]. Erastin, a highly known and effective ferroptosis inducer, targets xCT in tumors to induce ferroptosis [40]. Although many studies have suggested that cancer cells are resistant to Erastin and RSL3 induced cell death, Erastin is still a commonly used ferroptosis inducer in basic research [66]. Methionine, in addition to xCT, can be converted to cystine to synthesize GSH, hence preventing ferroptosis [67]. Two seemingly contradicting studies indicated differing functions of xCT in GBM and glioma stem cells (GSCs), respectively. According to one study, increasing the expression of xCT can improve GBM cell resistance to Gln/Glu starvation by decreasing ROS levels [68]. However, another study showed that, the overexpression of SLC7A11 induces DNA double-strand break (DSB) and enhances the sensitivity of GBM cells to radiotherapy [69]. According to the TCGA data, while the *SLC7A11* gene is highly expressed in GBM patients, it has a negative correlation with glioma WHO grades and the patients’ prognosis.

#### Transferrin Receptor

The stabilization of iron metabolism in brain is accomplished by a special set of proteins, one of which is TFR. Endothelial cells absorb ferrous iron via the Tf-TFR1 pathway, and these endothelial cells in brain constitute the BBB [70], which tightly regulates the movement of ions and molecules. Compared to normal brain tissue, GBM has a higher requirement for iron. GBM cells upregulates TFR1 to accommodate this high iron intake. Corresponding to this, TRF2 was also upregulated in GBM. The two members were dysregulated without sensing the intracellular iron level in tumor cells [71], resulting in the disruption of normal iron metabolic balance. On the basis of these, M.L. and N.H.L. et al. developed and evaluated Tf-functionalized pSiNPs (Tf@pSiNPs) with a perfect recognition in BBB cells and GBMs. Loading the anti-tumor drug doxorubicin (Dox) to Tf@pSiNPs system will significantly enhance the drug toxicity to U87-MG cell [69].

#### Tumor Protein 53

As mentioned above, tumor protein 53 (TP53) mediates ferroptosis occurrence by inhibiting cystine uptake. TP53 can upregulate the arachidonate 12-lipoxygenase12 (ALOX12) to suppress the growth of tumor cells through triggering ferroptosis [72]. TP53 can also enhance multienzyme activity to facilitate the accumulation of ROS. There are a variety of TP53 pathway-related protein mutations in GBM. The TP53 mutation is also one of the criteria used to classify glioma from low to high grades [73]. These findings also showed that the expression and mutation of TP53 can be used to evaluate the feasibility of treating GBM by inducing ferroptosis. Arachidonate lipoxygenase 3 (ALOXE3) promotes lipid peroxidation-mediated ferroptosis. The lack of ALOXE3 function causes GBM to produce resistance to TP53-dependent ferroptosis. The expression of ALOXE3 is considerably decreased in GBM, which induces the activation of the GRCRs, phosphatidylinositol 3 kinase (PI3K)—protein kinase B (AKT) pathways, enhancing GBM migration and invasion [74]. ALOXE3 interacts with SLC7A11 to inhibit the it. The miR18a, which has been proven to be upregulated in GBM, promotes GBM progression by blocking ALOXE3-mediated ferroptosis. ALOXE3 is the direct target of miR18a. The miR18a/TP53-ALOXE3/SLC7A11 axis provides an approach to suppress the growth of GBM cells via ferroptosis [75]. Rho family GTPase 1 (RND1) can deubiquitinate TP53 by directly interacting with it, enhancing the inhibited effect of TP53 on SLC7A11 [76]. The mutation of TP53 also determines SQSTM1 (P62)-NRF2-SLC7A11 axis-mediated ferroptosis in GBM. In TP53^wt^ GBM, P62 upregulates the expression of SLC7A11 through activating NRF2, exerting an anti-ferroptosis role. Conversely, in TP53^mt^ GBM, TP53^mt^ antagonizes the interaction of P62 and NRF2, inhibiting NRF2 downstream signals [77].

#### Nuclear Factor Erythroid 2 Related Factor 2

Nuclear factor erythroid 2 related factor 2 (NRF2) is encoded by *NFE2L2*. NRF2 is a pivotal transcription factor that regulates approximately 250 genes involved in maintaining cell homeostasis. Under normal physiological conditions, NRF2 binds to Kelch-like ECH associated protein 1 (KEAP1) and is inactivated through proteasome-mediated ubiquitination degradation. In contrast, when cells are subjected to oxidative stress, their interactions are disrupted, and NRF2 is subsequently shuttled to the nucleus [78]. In other words, Keap1 is sensitive to oxidative stress and acts as an NRF2 molecular switch. NRF2 regulates antioxidant response genes by binding their promoters to maintain a normal redox state. Hence, NRF2 activation can effectively eliminate ROS and have a detrimental impact on cancer treatment [79]. NRF2 regulates the transcription of three major groups of ferroptosis-related genes: iron metabolism genes such as *FTH1*, *HO-1*, and *FTL*,; ROS metabolism *PPARG*,; and the genes that regulate GSH synthesis such as *GPX4* and *SLC7A11* [80]. The upregulated expression of NRF2 in GBM tissue is obvious, and it is also related to a lower survival rate and poor prognosis [81]. It has been established that NRF2-KEAP1 is a regulator of xCT in GBM. NRF2 overexpression or KEAP1 knockdown can promote the malignant progression of GBM through ferroptosis-associated process [82]. Apolipoprotein C1 (APOC1) contributes to GBM-ferroptosis resistance in two ways. By inhibiting Keap1, APOC1 boosts NRF2’s nuclear transportation and increases the expression of HO-1. Another, effect of APOC1 was an increase in cystathionine beta-synthase (CBS) expression, which promoted trans-sulfuration and increased GSH synthesis, resulting in a rise in GPX4 expression [83]. Ibuprofen (NSAID), a traditional nonsteroidal anti-inflammatory drug, has been shown to have anti-tumor functions in GBM by inducing NRF2-mediated ferroptosis. NSAID decreases NRF2 protein expression, downregulating the mRNA levels of GPX4 and SLC7A1 [84]. On the basis of previously reported Chalcone analogues-CET-CH-1 to CET-CH-5, R.A. and N.S., et al. developed CET-CH-6, which is a novel RNF2 inhibitor. Surprisingly, CET-CH-6 exerts RNF2 inhibitory function in TP53 (R175H) mutant GBM cells [85].

#### Acyl‑CoA Synthetase Long‑chain Family Member 4

The downregulation of Acyl‑CoA synthetase long‑chain family member 4 (ACSL4) has been linked to ferroptosis and GBM proliferation. Overexpression of ACSL4 in a GBM cell line was shown to downregulate GPX4 while increasing the expression of relevant ferroptosis markers [86]. Heat shock protein (HSP) family members are known to interact with various proteins that have mutation and tumorigenic functions. HSP27 interacts with ACSL4 to modulate its stability, increasing ferroptosis-resistance in GBM cells [87]. Dihydrotanshinone I (DHI) has been shown to have an effect on the expression of ferroptosis-related proteins in GBM, which downregulates GPX4 and intracellular GSH levels by increasing the expression of ACSL4 [88].

#### Heme Oxygenase-1

*HOMX1* encodes heme oxygenase-1 (HO-1), which affects the iron cycle and ROS. HO-1 has been suggested to eliminate peroxy free radicals and decrease lipid peroxidation, hence reducing intracellular oxidative stress [89]. The expression of *HOMX1* is significantly negatively associated with the survival of GBM patients [90]. High expression of HO-1 accelerates the invasion and migration of GBM cell lines in vitro [91]. Siramesine, a lysosomotropic agent, and lapatinib, a dual tyrosine kinase inhibitor (TKI), can synergistically increase the level of ROS and accumulation of lipid peroxide in GBM, both of which are indicators of ferroptosis. Importantly, when two drugs are combined, there is a clear downregulation of HO-1 [92]. Interestingly, another study in neuroblastoma (NBL) showed that withaferin A can over-activate HO-1 by targeting Keap1 and GPX4, resulting in increased LIP and triggering nonclassical ferroptosis [93]. Thus, in different types of tumors, HO-1 has opposing “dark” and “bright” effects. On the one hand, HO-1 can either promote or inhibit tumor progression by affecting the degree of angiogenesis and tumor metastasis. On the other hand, overexpression of HO-1 causes ER stress, mitochondrial dysfunction, and an increase in intracellular LIP [94]. Clearly, HO-1 is extremely complex in terms of its tumorigenesis promotion and inhibition, but it may be a viable target for GBM treatment, that is, designing targeted inhibitor of HO-1 “dark” side based on its elevated expression in GBM.

### Ferritin Autophagy and Endoplasmic Reticulum Stress in GBM Ferroptosis

Autophagy is also involved in ferroptosis. Ferritin is an iron storage protein that is mostly synthesized in the liver [95]. Nuclear receptor coactivator 4 (NCOA4) recognizes ferritin and forms a complex with it. This complex will later fuse with the lysosome, leading to ferritin degradation and the release of free irons [96]. The coat complex subunit zeta 1 (COPZ1) is overexpressed in GBM and can block ferritin autophagy by inhibiting NCOA4. Downregulating the expression of COPZ1 in U-87MG and U251 cells induces ferritin autophagy and increases intracellular iron levels, eventually leading to ferroptosis [97]. Amentoflavone (AF) treatment of GBM cells increases the expression of autophagy-related proteins LC3B, Beclin-1, and ATG5/7, promoting autophagic degradation of FTH and causing ferroptosis [98]. Cysteine deficiency can cause ferritin degradation via autophagy mediated by LC3B and NCOA4, resulting in ferroptosis in GBM. However, the glutamine levels are necessary for ferroptosis induced by cysteine deprivation [99]. Simultaneously combining autophagy and ferroptosis inducers may be a promising option for GBM clinical treatment.

It is widely accepted that ferroptosis is caused by excessive ROS accumulation. Excessive ROS, on the other hand, disrupt endoplasmic reticulum homeostasis, resulting in ER stress [100]. Activating transcription factor 4 (ATF4) functions as a key molecule in ER stress and is mainly regulated by the protein kinase R (PKR)-like endoplasmic reticulum kinase (PERK)- eukaryotic translation initiation factor 2 subunit alpha (eIF2α)-ATF4 pathway [101]. Later, ATF4 upregulates the C/EB homologous protein (CHOP), which enhances the expression of proapoptotic proteins, such as P53 upregulated modulator of apoptosis (PUMA) and BCL-2 interacting mediator of cell death (BIM). A previous study demonstrated that erastin can upregulate the ER stress-related-molecule ChaC glutathione specific gamma-glutamyl-cyclotransferase 1 (CHAC1), a downstream member of ATF4. CHAC1 promotes GSH decomposition and trigger ferroptosis [102, 103]. ATF4 is highly expressed in the tissues of GBM patients and has a positive correlation with glioma WHO grades. In GBM, ATF4 can promote *SLC7A11* transcription, whereas erastin and RSL3 can reverse this effect. Hence, the crosstalk between the ER stress and ferroptosis pathways, as well as the coregulator ATF4, may be a potential target for GBM treatment [104]. Dihydroartemisinin (DHA), a Chinese patent herb, has anti-tumor effects by enhancing ROS levels. On the other hand, DHA can reversely activate ER stress to avoide ferroptosis. DHA specifically upregulates ATF4 and then induces the expression of the endoplasmic reticulum chaperones BiP and GPX4. Small molecule inhibitors can be used to inhibit the ATF4-HSPA5-GPX4 axis and combined with DHA will enhance the sensitivity of GBM to ferroptosis [105]. ATF3, a downstream molecule of the PERK-ATF4 axis, promotes the production of H_2_O_2_ by upregulating NOX4 and SOD. Brucine, a weak alkaline indole alkaloid extracted from the seeds of Strychnos nux-vomica, can increase the nuclear transport of ATF3 by activating ER stress, promoting ferroptosis in GBM [106].

### Ferroptosis and TMZ Chemotherapy

To date, TMZ, a standard first-line chemotherapeutic treatment for GBM, has been shown to greatly improve the median survival time for GBM patients. However, in recent years, the subsequent TMZ resistant problem have also emerged in GBM treatment. Although the related mechanisms have been studied extensively, the challenge remains intractable [107]. Growing evidence suggests the relationship between ferroptosis and TMZ resistance. Androgen receptor (AR) is negatively correlated with the prognosis of GBM patients and was proved to induce resistance to TMZ chemotherapy. ALZ003, a curcumin analog, can increase AR ubiquitination by increasing the expression of F-box and leucine-rich repeat protein 3 (FBXL3), an E3 ubiquitin ligase, resulting in the degradation of AR. ALZ003 can boost ROS levels even further, inhibit GPX4 to trigger ferroptosis, and recover TMZ cytotoxicity. More importantly, AR overexpression blocks ferroptosis in GBM [108]. Long-term TMZ treatment has also been linked to ferroptosis resistance in GBM by upregulating xCT. When the xCT inhibitor or siRNA knockdown is used, TMZ activates cystathionine γ-lyase (CTH), a crucial enzyme in the transsulfuration pathway, to ensure the availability of cysteine and GSH [109]. Overexpression of xCT can also enhance the GBM tolerance to sulfasalazine (SAS), a drug approved by the Food and Drug Administration (FDA) and European Medicines Agency (EMA) for GBM [110]. In addition, GPX4, NRF2 [104], ATF4, and TP53 may have effects on the TMZ resistance of GBM [111].

### Ferroptosis-inducing Compounds in GBM

Recently, various ferroptosis-inducing compounds (FINs) have been developed. These FINs are divided into the following types based on their ability to target specific ferroptosis-associated proteins. The first are xCT subunit inhibitors like erastin and its analogs, followed by GPX4 inhibitors, such as RSL3 and FINO2 [112] and finally agents that impact the iron transport balance and increase LIP levels [94].

In Table 1, we summarized the FINs that have been reported for GBM treatment. These FINs findings also indicate that targeted ferroptosis is a promising GBM treatment strategy. Furthermore, an innovative GBM chemotherapy that locally targets ferroptosis was reported last year. Zhang et al. designed iron oxide nanoparticles (IONPs), that can simultaneously deliver GPX4-targeting siRNA and cisplatin (Pt), a common clinical chemotherapeutic drug, to the GBM lesion region. The PT in the INOPs induces apoptosis by breaking mitochondrial DNA and increases the hydrogen peroxide content by activating NOX. Meanwhile, siGPX4 RNA assists in inducing ferroptosis in U-87MG and P3# GBM cell lines [113].

**Table 1:** The list of ferroptosis-inducing compounds

FINs	Targets	Mechanisms
Pseudolaric acid B (PAB) [56]	TFR, NOX4, TP53	Inhibit the xCT and upregulate the TFR to activate NOX4
Dihydroartemisinin (DHA) [105]	ER stress pathway and GPX4	Activate both pathways that promote and inhibit ferroptosis
ALZ003 [108]	Androgen receptor (AR)	Downregulate the expression of GPX4 and lead to the ROS accumulation
IONPs [113]	GPX4 and NOX	Nanoparticle IONPs use the carried siGPX4 to target and inhibit GPX4, and the carried cisplatin can disrupt mitochondrial function and increase the ROS level
Dihydrotanshinone I (DHI) [88]	ACSL4 and GPX4	Downregulate the ACSL4 and GPX4, at the same time decrease intracellular GSH level
Amentoflavone (AF) [98]	FTH, LC3B, Beclin1	Decrease the expression of FTH by inducing autophagy
Brucine [106]	ATF3	Induce the ER stress and promote the expression and nuclear transport of ATF3, thereby upregulating NOX4 and suppressing xCT
Ibuprofen (NSAID) [84]	Nrf2, xCT, GPX4	Decrease the expression of Nrf2 and prevent the cystine transfer, bringing about ROS accumulation
Disulfiram (DFS) [63]	xCT and GPX4	Downregulate the expression of xCT and GPX4, at the same time enhance the ROS level

**Fig. 2:**
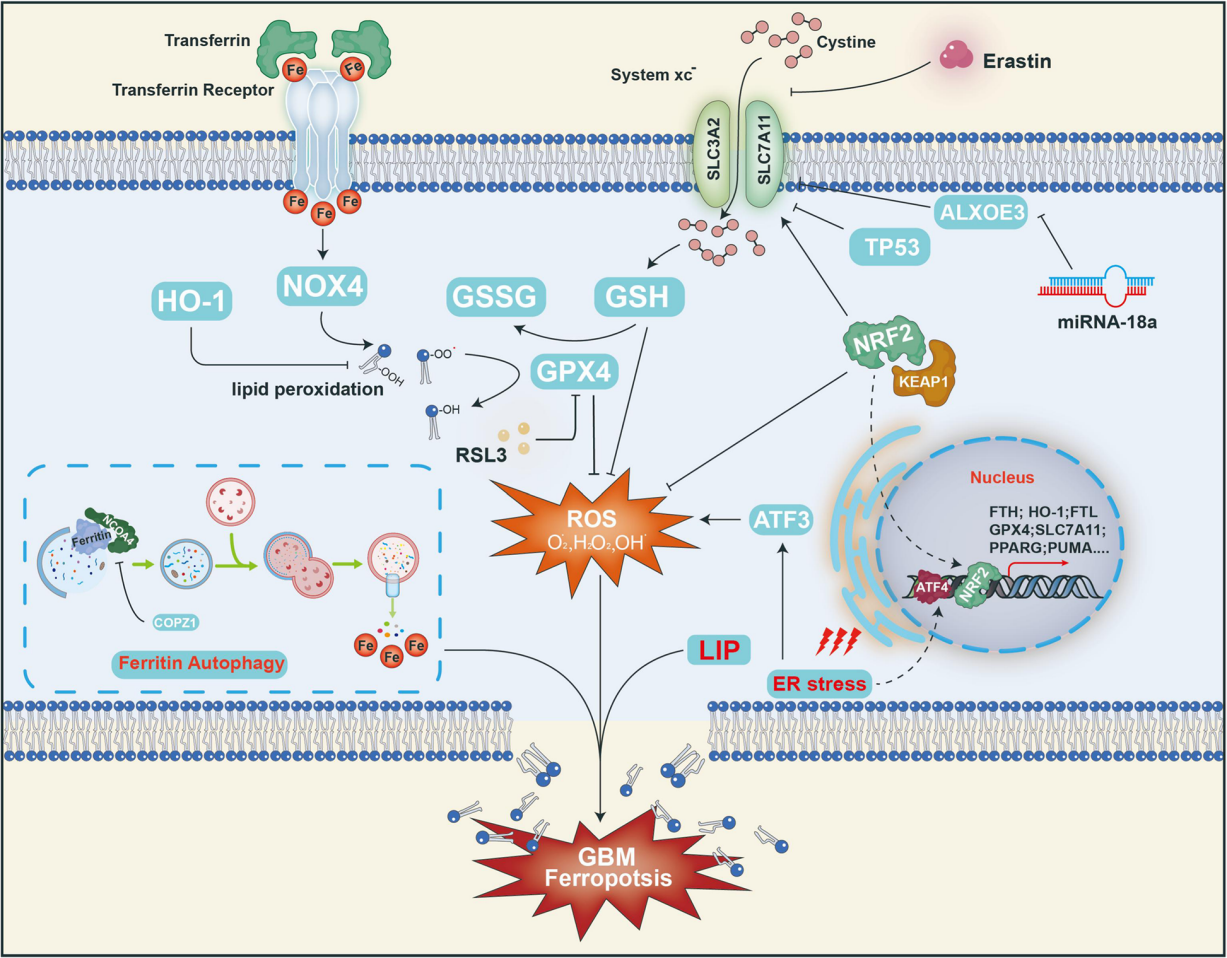
The regulatory mechanism of ferroptosis in GBM

Ferroptosis-resistant genes such as *GPX4*, *TFR2*, *NFE2L2*, *KEAP1,* and *HMOX1,* etc., show significantly higher expression in GBM compared to normal brain tissues (Supplementary Fig. 1B), implying abnormal iron metabolism in GBM. As shown in Fig. 2, ferroptosis is tightly regulated by many molecules and is intimately related to iron metabolism and oxidation homeostasis in GBM. Because GBM has evolved a range of abnormal metabolic pathways in response to the changing TME, it is possible to design ferroptosis-related core molecular inducers to reverse these metabolic processes. In addition, the combination of FINs and traditional chemotherapeutics has the potential to be a novel approach for clinical GBM therapy.

## Autophagy and Glioblastoma

Autophagy, also known as PCD type II, has been extensively studied, and in terms of its effect on cellular fate, it differs from other PCDs. Autophagy plays a key role in maintaining cell survival under stress, only in rare cases does it results in cell death.

### Regulatory Mechanisms of autophagy

Autophagy is a conserved biological process in prokaryotes and eukaryotes that helps in the turnover of intracellular substances [114]. Autophagy is divided into three categories based on how substrates to be degraded enter the lysosome: microautophagy, macroautophagy, and molecular chaperone-mediated autophagy (MCA) [115]. Macroautophagy has been extensively investigated, and it involves damaged protein aggregates and organelles being packaged by autophagosomes and then fusing with lysosomes. Finally, acidic hydrolases in lysosomes digest these cellular trashes, and the degraded products are re-released into the cytoplasm, where they are re-used as nutrients by cells. The complete process involves four independent steps: the formation of preautophagosomal structure (PAS) or omegasome, the formation of autophagosomes, the autophagosome-lysosome fusion, and the release of autophagosome contents after hydrolysis [116–118].

A number of molecules participate in the entire autophagy process. For example, the FIP200, ATG13, ATG101, and ULK1 complex (ATG1) connect upstream nutrition or energy receptors and downstream autophagosomes. Upstream elements of ULK1 are the mammalian target of rapamycin (mTOR) and adenosine 5 ‘-monophosphate (AMP)-activated protein kinase (AMPK). Its downstream complex, the vacuolar protein sorting 34 (VSP34) complex, the III type PI3 Kinase (PIK3C3) in mammals, can phosphorylate phosphatidylinositol (PI), the main component of the cell membrane, and produce phosphatidyl inositol triphosphate (PI3P). PI3P is important for autophagic bubble membrane extension and ATG recruitment to PAS. MAP1LC3-II (LC3-II, ATG8) and the phosphatidyl ethanolamine (PE) complex can bind to autophagosome membranes and help in membrane extension. The complex is also a structural protein of autophagosomes, and its level is a crucial molecular marker for determining autophagy flux.

Autophagy is initiated and inhibited by two intracellular energy sensors, mTOR and AMPK. mTOR can integrate changes in the deficiency of intracellular amino acids, hypoxia, and other cues to regulate the occurrence of autophagy. mTOR inhibits autophagy by phosphorylating ULK1 under normal nutrition. In the absence of energy, the cell activates AMPK by sensing an increase in the ratio of AMP to ATP. At this point, AMPK will directly phosphorylate ULK1 to initiate autophagy. When there is enough intracellular energy, mTOR inhibits ULK1 by phosphorylating its different amino sites, suppressing autophagy [117–121].

Generally, tumor cells require adequate metabolism to sustain their proliferation capacity, hence, they have considerably higher energy and nutritional requirements than normal cells [122]. Tumor cells under nutrient deficient TME activate protective autophagy to help them survive the crisis. For instance, after chemoradiotherapy, large amounts of damaged organelles and proteins are produced. Increased autophagy activity helps cells remove this “trash” providing energy, emergency substrates, and time for tumors to repair damaged DNA. Based on this, NSC185058, an ATG4B inhibitor, can greatly enhance the effect of radiotherapy in GBM clinical treatment [123].

However, it has also been suggested that autophagy mainly mediates the cleanup of cells killed by replication crises, hence inhibiting the initiation of tumorigenesis early [124]. Collectively, autophagy is a double-edged sword in multiple cancers, including GBM [125–127].

### Hypoxia, a New Player in GBM Protective Autophagy

#### Molecules Related to Hypoxia-Induced GBM Autophagy

The hypoxic GBM TME will induce autophagy [128]. The transcription factor Hypoxia inducible factor-1α (HIF-1α) is closely related to cell hypoxia. Under normal oxygen conditions, HIF-1α and endothelial PAS domain-containing protein 1 (EPAS1, HIF-2α) are hydroxylated by the prolyl hydroxylase EGLN (EGLN) family members and then recognized and degraded by E3 ubiquitin ligase, von Hippel-Lindau disease tumor suppressor (VHL). Under hypoxic conditions, hydroxylase is inactivated, leading to the accumulation of HIF-1α and EGLN and the induction of survival gene transcription to adapt to the hypoxic environment. The expression of HIF-1α and EGLN is increased in many types of cancer, which is generally related to a poor prognosis [129]. Histanoxia has been shown to activate HIF-1α and promote autophagy. Hypoxia-induced autophagy contributes to the construction of vasculogenic mimicry (VM) to maintain cell proliferation by activating the PI3K-AKT signal [130]. The p21 [RAC1] activated kinase 1(PAK1) acts as a hypoxia-induced positive regulator of protective autophagy, accelerating GBM cell proliferation. Hypoxia increases PAK1 activity, which protects ATG5 from ubiquitination degradation and subsequently promotes autophagosome formation [131]. In GBM, the Myc proto-oncogene protein (c-MYC), a known transcription factor, can regulate the Ras-Associated Protein 7 (Rab7a) expression and is activated by hypoxia. The expression of Rab7a initiates the protective autophagy in GBM, preventing apoptosis [132]. Rab7a controls autophagic flux by switching active GTP and inactive GDP binding states on autophagosomes [133]. Hypoxia influences the expression of miR-224-3p, which induces protective autophagy in GBM [134]. The autophagy-related protein Beclin1 can be activated via phosphorylation by HIF-1α to generate protective autophagy in GBM [135]. In the necrotic hypoxic tissue of GBM, autophagy is activated by BCL-2 interacting protein 3/like (BNIP3/BNIP3L), which is acted as a survival mechanism of GBM cells by promoting their resistance to chemotherapeutic drugs [136]*.* The *Retinoblastoma gene* (*Rb)* is a well-known tumor suppressor gene that encodes the retinoblastoma tumor suppressor protein (Rb), and its deletion and mutation are related to poor prognosis. Its downstream member, E2F transcription factor 1 (E2F1), can regulate the expression of autophagy-related genes [137]. The Rb-E2F1 axis regulates the expression of BNIP3 in the hypoxic GBM TME, as mentioned above BNIP3 is essential for hypoxia-mediated protective autophagy [138].

#### Bevacizumab and Hypoxia-Induces GBM Autophagy

Routine chemoradiation therapy for GBM combined with bevacizumab (BVZ), a vascular endothelial growth factor (VEGF) inhibitor, can improve the PFS and overall survival (O.S.) of patients [139]. Recent studies suggest that BVZ induces hypoxia, which leads to protective autophagy in GBM by suppressing the AKT-mTOR pathway and VM [140]. Two groups of researchers have suggested that the knockdown of ATG7 and ATG9A will rescue GBM sensitivity to BVZ [141–143].

#### Isocitrate Dehydrogenase and Hypoxia-Induces GBM Autophagy

The isocitrate dehydrogenase 1 and 2 (IDH1/2) mutation is a vital molecular marker for GBM classification. IDH1 mutation (IDH^mut^) cases comprise to 80% of all low-grade GBM (II-III) and is associated with prolonged patients’ survival [144]. IDH^mut^ is linked to hypoxia, angiogenesis, and HIF-1α expression, all of which are key autophagy initiating elements [145]. The autophagy level in IDH^mut^ GBM is lower than that in IDH wild type (IDH^wt)^. The expression of LC3-II, Beclin1, and P62 is also higher in IDH^wt^ than in IDH^mut^ GBM [146, 147].

Overall, hypoxia-induced autophagy is associated with a number of complex factors, such as heredity, metabolism, and the TME. Inhibiting autophagy, induced by hypoxia, may become an effective GBM clinical treatment. Hypoxia-related biomarkers could be a new research direction in the regulation of autophagy in GBM.

### EGFR Mutation in GBM Autophagy

The epidermal growth factor receptor (EGFR) is associated with a variety of molecules and pathways involved in cell proliferation, and it is widely utilized as a pathological diagnostic marker for many types of cancer. The mutation, amplification, and rearrangement of *EGFR* contribute to GBM invasion and metastasis. The most common EGFR mutant form in GBM is EGFRVIII. *EGFR VIII*, without the 2–7 exons of *EGFR*^*wt*^, encodes a short extracellular domain that is independent of EGF interaction and consistently activates its downstream signaling. A number of previous studies have demonstrated that EGFRVIII affects the progress of tumors by regulating the Ras-Raf-MEK-ERK-MAPK axis and is the main factor for tumors escaping chemoradiation therapy [148–150]. The hypoxic environment will activate protective autophagy in EGFRVIII-GBM, luckily patients can get benefit from synergistic treatment with chloroquine (CQ) [151], which blocks autophagy by inhibiting autophagosome-lysosome fusion and subsequent lysosome degradation [152]. Tumor suppressing subtransferable candidate 4 (TSSC4) is highly expressed in EGFRVIII-GBM, inhibits autophagy by interacting with LC3, preventing GBM overgrowth. In contrast, when TSSC4 level is low, LC3 does not bind to TSSC4, increasing the level of autophagy, leading to excessive cell proliferation [153]. The 4-Hydroxy tamoxifen (OHT), an active metabolite of the tamoxifen (TMX) prodrug, can induce cytotoxic autophagy in EGFR^wt^ GBM cells, inducing cell death, but EFRVIII-GBM cells will gradually hold tolerance to OHT after long-term treatment [154]. Tyrosine Kinase inhibitors (TKIs) like gefitinib and erlotinib combined with CQ produce a better anti-tumor effect in the clinic. Furthermore, GBM patients who have low EGFR expression but high Beclin1 expression imply a better prognosis [155, 156].

### Classical Signaling Pathways Related to GBM Autophagy

#### PI3K/mTOR and TP53 Pathway

Extracellular signals suppress the PI3K/AKT/mTOR signaling cascade in stressful environments such as hypoxia or nutrient depletion, leading to the activation of protective autophagy. This avtivated cascade can be observed in a number of clinical GBM samples due to the overexpression of upstream EGFR or inactivating mutations in PTEN [157]. The PI3K inhibitor treatment inhibits GBM invasion and angiogenesis, but it will also activate autophagy. But, overall, PI3K suppression effectively limits GBM proliferation and significantly prolongs the survival time of tumor-bearing mice [158].

TP53 and its pathways are considered to be tumor migration, invasion, and proliferation regulators, and their dysfunction results in a poor prognosis. Recent studies suggest that TP53 in the nucleus mediates *ATGs* expression through transcriptional regulation. But, then TP53 in the cytoplasm can inhibit protective autophagy. GSCs, a subgroup of glioma cells, can self-renew and are often associated with postoperative recurrence in GBM patients [159]. Nucleus-TP53 can upregulate the expression of DNA damage regulated autophagy modulator 1 (DRAM1), which acts as an activator of autophagy and promote the migration and invasion of GSCs.

#### Wnt and Sonic Hedgehog Pathway

Wnt signaling regulates many biological processes, such as cell proliferation, adhesion, and movement. Members of the Wnt pathway play a key role in GBM invasiveness and drug resistance. Wnt signaling inhibition suppresses autophagy by downregulating LC3 and Beclin1. DOC-2/DAB2 interacting protein (DAB2IP) downregulates ATG9B by inhibiting the Wnt pathway, hence enhancing TMZ cytotoxicity [160]. Suppressing the Wnt-CTNNB-β-catenin axis will upregulate P62, which is associated with the blockade of autophagy. Inhibiting this axis can increase the sensitivity of cells to CQ or other autophagy inhibitors [161]. Additionally, the miRNA let-7 g-5p can inhibit the activation of Wnt-β-catenin pathway and promote apoptosis and autophagy [162]. Conversely, autophagy leads to the attenuation of the Wnt pathway and the re-localization of β-catenin in GBM cells. When autophagy is inhibited, β-catenin is located in the nucleus and interacts with transcription factors (TCFs) to drive the transcription of downstream target genes. When autophagy is activated, β-catenin binds to N-cadherin to form epithelial-like cell–cell adhesion structures on the GBM cytomembrane, hence contributing to epithelial-mesenchymal transition (EMT) [163].

The sonic hedgehog (Hh) signaling pathway is associated with multiple tumorigenic pathways, including the PI3K-AKT-mTOR pathway [164]. The Hh pathway can inhibit or activate autophagy by regulating different intermediate molecules [165]. However, almost all of the recent studies on GBM reported that inhibition of the Hh pathway negatively regulated the tumor cells proliferation by activating autophagic death. GANT-61, a Hh signaling inhibitor, has been shown to elevate TMZ toxicity by activating cytotoxic autophagy in TMZ-resistant GBM cell lines [166, 167]. Another Hh signaling inhibitor, LDE225, has been suggested to induce autophagic death of GSCs in a mTOR independent manner [168]. Compared with primary GBM tissues, high SRY-Box transcription factor 3 (SOX3) expression was detected in recurrent GBM tissues, and the exogenous overexpression of SOX3 in GBM cells can enhance the activity of Hh signaling and inhibit cytotoxic autophagy, increasing migration and invasion capabilities [169].

In GBM, YAP, a key factor in the Hippo signaling pathway, can induce protective autophagy. YAP overexpression upregulates the expression of high mobility group protein B1 (HMGB1), and trigger protective autophagy, and this process can be blocked by CQ [170]. The *Rho family GTPase 2* (*RND2*) has been discovered as an oncogene; its coding protein interacts with p38 MAPK and inhibits its phosphorylation, hence blocking the MAPK signaling pathway. Moreover, RND2 overexpression in U251MG and U-87MG cells inhibits apoptosis and cytotoxic autophagy in a MAPK inhibition-dependent manner [171]. Finally, several studies have also found a correlation between the Notch signaling pathway and GBM cytotoxic autophagy. Silencing the Notch1 receptor in GBM can decrease cell proliferation by upregulating Beclin1 and LC3-II [172]. In line with this, mTOR inhibition will lead to the autophagic degradation of the Notch1 receptor, reducing the tumorigenicity of GSCs [173].

### Frontiers in Drug Studies Related to GBM Autophagy

#### Autophagy and TMZ Chemotherapy

Long-term doses of TMZ have been demonstrated to induce protective autophagy in GBM cells [174]. The combined utilization of TMZ with autophagy-related molecular inhibitors in GBM research, and the protective autophagy mechanism caused by TMZ have attracted considerable attention.

When combined with TMZ, a kinase inhibitor regorafenib has a better inhibitory effect on GBM cell. Regorafenib physically binds to phosphoserine aminotransferase 1 (PSAT1), triggers AMPK-mediated autophagy initiation, and negatively regulates Ras-related protein Rab-11A (RAB11A)-mediated autophagosome-lysosome fusion. Such simultaneous autophagy initiation and inhibition lead to the accumulation of a large number of autophagosomes in GBM, resulting in lethal stagnation of autophagy, which leads to the autophagic death of GBM cells [175]. LCRR4 has been identified as a new autophagy suppressor that can restore GBM sensitivity to TMZ. Specifically, LCRR4 binds to the DEPTOR/mTOR complex and reduce its half-life through ubiquitination degradation [176]. It has also been shown that DNA damage-inducible transcript 4 (DDIT4) induces autophagy in TMZ-resistant GBM cells. After TMZ treatment, ATF4 upregulates the expression of DDIT4. On the one hand, the ATF4-DDIT4 axis leads to protective autophagy. DDIT4, on the other hand, also upregulates glucose transporter-3 (GLUT3) in GBM, which participates in the stemness maintenance of tumor cells and reduces TMZ cytotoxicity [177]. Continuous TMZ treatment will promote the production of GSCs and the secretion of PD-L1 containing exosomes (PD-L1-ex). PD-L1-ex inhibits cell apoptosis and induces protective autophagy by activating AMPK/ULK1-mediated autophagy, eventually resulting in TMZ resistance [178]. Glioma initiating cells (GICs), a type of primary cell isolated from the glioma, have the ability to regenerate into complete tumor. GICs have been considered the initiating factor of glioma occurrence, proliferation, metastasis, and resistance to chemoradiation therapy [179]. Research on GICs has shown that the neurotrophic factor MIDKINE (MDK) can promote TMZ resistance in GBM. Inhibition of MDK and its ALK receptor tyrosine kinase (ALK) can reduce the proliferation of GICs by promoting the autophagic degradation of SRY-Box transcription factor 9 (SOX9), a transcription factor. Moreover, blocking this pathway combined with TMZ will reduce GICs’ tumorigenesis in xenograft mice [180].

#### Other Advances of Compounds and Drugs in GBM Autophagy

Many other drugs and small molecular inhibitors associated with GBM autophagy have also been identified, for example, pimozide, a drug originally used to treat mental illness, exerts anti-tumor function in GBM by inducing autophagy-mediated apoptosis [181]. Lysosomal membrane permeabilization (LMP) has the potential to cause lysosome-dependent cell death (LDCD). LMP causes the lysosomal membrane to lose its integrity and release the contents into cytoplasm, and this process can be induced by lysosomotropic agent [182, 183]. The lysosomotropic agent alys05, shows effective autophagy inhibition and anti-glioma effects in vitro. Another study confirmed that the combination of lpoeramide and pimozide triggers LMP by inhibiting the activity of sphingomyelin phosphodiesterase 1 (SMPD1), which affects the transport of lipids and cholesterol to lysosomes [184]. The gum resin component 3-O-Acetyl-11-keto-β-boswellic acid (AKBA) can block abnormal autophagy in orthotopic GBM mice model by regulating ERK/mTOR and P53/mTOR pathways [185]. Platycodin D (PD), extracted from Latycodon Grandiflorus (PG), can impede autophagosome-lysosome fusion, thereby inhibiting the protective autophagy in GBM. At the same time, PD can also disrupt cholesterol transport in GBM by up-regulating the low-density lipoprotein receptor (LDLR), causing the accumulation of cholesterol in the lysosome and triggering LDCD [186]. Table 2 summarizes the drugs or small molecular inhibitors that inhibit cytoprotective autophagy or induce cytotoxic autophagy to exert anti-glioma function.

**Table 2:** The list of chemotherapeutic agents and their target proteins or pathways in GBM

Types	Drug	Targeted proteins or pathway	Main mechanisms
Inhibit cytoprotective autophagy	Regorafenib [175]	PAST1, AMPα, RAB11A	Inhibit the autophagosome-lysosome fusion
	Pimozide and Loperamide [181]	ATG5, ATG7, Sphingomyelin Phosphodiesterase 1 (SMPD1)	Lead to the lysosomal membrane permeabilization
	Lysosomotropic agent: Lys05 [182]	LC3-II, P62	Lead to the lysosomal membrane permeabilization
	Platycodin D (PD) [186]	LC3-II, P62, LDLR, cathepsine B (CTSB)	Prevent the autophagosome-lysosome fusion and inhibit the function of lysosomes
	Simvastatin and TMZ [187]	eIF2α, PERK	By triggering U.P.R. to induce eIF2α phosphorylation, thereby activating ATF4 transports into the nucleus and inhibits the transcription of related autophagy target genes
	3-O-Acetyl-11-keto-β-boswellic acid (AKBA) [185]	ATG5, P62, LC3-II, ERK, P53	Improve the abnormal metabolism in GBM through ERK-mTOR and P53-mTOR to inhibit autophagy
Induce cytotoxic autophagy	GANT-61 [166, 167]	Hh pathway, Beclin1	Inhibit Hh pathway, upregulate Beclin1, and enhance the ROS level
	Lactucopicrin [188]	p-AKT, p-ERK, CDK2, P53, P21	Decrease the phosphorylation of AKT and ERK, activate autophagy and induce G2/M cycle arrest
	Nanomicellar-Curcumin [189]	Beclin1, LC3-II, Wnt pathway	Upregulate autophagy-related genes and downregulate Wnt pathway

**Fig. 3:**
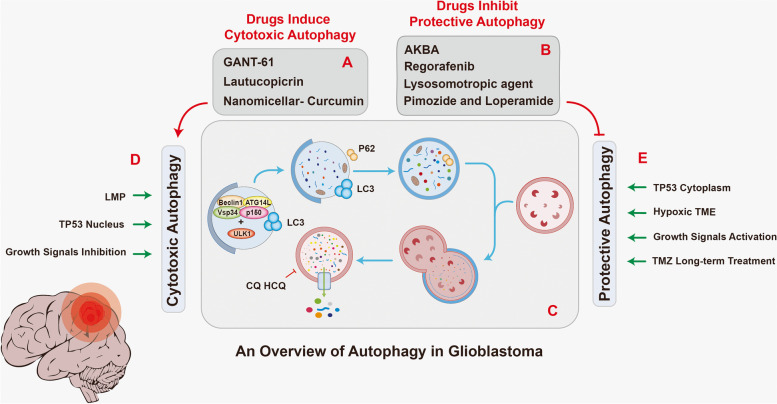
The molecular basis of autophagy and its regulatory mode in GBM. Autophagy induced by drugs or stress in GBM can be divided into two types: cytotoxic autophagy and protective autophagy. Usually, the former exerts negative impact on the growth of GBM, while the latter is just the opposite, and as shown in (**A**) and (**B**), the two types of autophagy can be respectively induced and inhibited by drugs. In addition, as the (**D**) and (**E**) show, the occurrence cytotoxic autophagy and protective autophagy is related to a variety of factors, such as LMP induced by lysosomotropic agents, hypoxic TME, and nuclear or cytoplasmic localization of TP53, etc. Finally, (**C**) shows the whole process of autophagy, which can be blocked by CQ and HCQ

Through retrospecting the previous studies, we concluded that the dual roles of autophagy in GBM are the results of changes in cell homeostasis caused by chemoradiation therapy and inhibitors (Fig. 3). While, a portion of autophagy-related genes are also highly expressed in GBM (Supplementary Fig. 1C), this is insufficient to be considered an independent indicator of autophagy status. The explanation for this is that autophagy is largely determined by the extracellular environment (osmotic pressure, hypoxia) and the intracellular nutritional state.

It is still unknown how to correctly induce cytotoxic autophagy and block protective autophagy in GBM. The epigenetic modification of autophagy-related genes and other modifications (phosphorylation, ubiquitination, and acetylation) in their coding proteins are regulated by multiple signaling pathways in GBM. Therefore, it is necessary for researchers to utilize multi-omics approaches to screen abnormally amplified and mutated autophagy-related genes in GBM. In addition, discovering novel autophagy transduction pathways and developing new drugs or inhibitors based on them could be greatly beneficial to GBM research.

## Crosstalk Between Apoptosis, Pyroptosis, Ferroptosis and Autophagy

With the large-scale clinical application of apoptosis targeting drugs, many types of cancer, including GBM, have evolved strategies to evade drug-induced apoptosis. Regarding the apoptosis mechanisms in GBM or other cancers, many excellent studies have been published, so we do not go into detail here [190–192]. The crosstalk among pyroptosis, ferroptosis, autophagy, and apoptosis is particularly noteworthy. It is apparent that members of the caspase family can induce both pyroptosis and apoptosis. In lung cancer [193] and melanoma [194] the caspase-3/GSDME axis is used as a switch between apoptosis and pyroptosis. Under the activation of external pathogens, in macrophages lacking GSDMD/E, caspase-1 and caspase-8 mediate the cleavage of the BH3-interacting domain death agonist (Bid), leading to the release of cytochrome C from mitochondria, and the subsequent apoptosis cascade [195]. However, studies have shown that isobavachalcone (IBC), a natural prenylated chalcone compound, induces mitochondria-dependent apoptosis in GBM while alleviating pyroptosis [196]. ROS and lipid peroxidation are linked to not only ferroptosis but also the unfolded protein response (UPR) and ER stress. ER stress induces the nuclear transport of CHOP, leading to the expression of PUMA, growth arrest, DNA damage–inducible protein (GADD34), and other apoptosis-related proteins at the transcriptional level. Synergy between ferroptotic agents and the apoptotic agent tumor necrosis factor-related apoptosis-inducing ligand (TRAIL) has also been found to mediate this process [100]. Furthermore, TP53 acts as a mediator between apoptosis and ferroptosis. The connection between autophagy and apoptosis is widely recognized, and the relationship between them can be generalized into two types: cooperation and antagonism. Firstly, autophagy can collaborate with apoptosis to trigger cell death, which often occurs in cytotoxic autophagy in chemotherapy. Secondly, autophagy, as the maintenance mechanism for cell survival, makes cancer cells escape from apoptosis, which often can be seen in drug-resistant tumors [197].

In terms of the correlation between ferroptosis and autophagy, lipophagy [198] and the xCT autophagic degradation mediated by Beclin-1 [199] were found to trigger ferroptosis. Recently, several studies provided evidence that autophagy is the medium of external factors induced ferroptosis, especially ferroptosis inducers. Autophagy tends to trigger ferroptosis by regulating intracellular levels of ROS, LIP, and lipid peroxides [200]. Such a manner is even known as the autophagic ferroptosis, which strengthens their connection, despite the fact that these studies primarily focus on other disease models rather than glioma. The findings on crosstalk mainly focus on the system xc^−^, GPX4, and FTH. After being phosphorylated by AMPK, Beclin-1 will form a complex with SLC7A11 and inhibit its function of Cys transport, promoting lipid peroxidation and ferroptosis [201]. Paraoxonase 1 (PON1) elevated Glu level by autophagy-mediated decomposition of intracellular substance, in turn, activating system xc^−^ to transport Cys into intracellular space. PON1 also makes cells develop resistance to ferroptosis by impacting TP53-SLC7A11 [202]. The *O*-GlcNacylayion of FTH will evade NCOA4-mediated FTH degradation and prevent cells from ferroptosis [203]. Conversely, zinc oxide nanoparticles (ZnONP) promote autophagic degradation of FTH through activating the AMPK-ULK1 axis [204]. Similarly, it has also been shown that GPX4 is degraded by interacting with different autophagic receptors after Fin56 (type 3 ferroptosis inducer) or excessive copper treatment, triggering ferroptosis [205, 206].

The relationship between pyroptosis and autophagy, is mainly related to P62 and NLRP3. P62 can ubiquitinate damaged mitochondria and trigger mitophagy to avoid activating caspase-1 [207]. NLRP3 activation, on the one hand, eliminates damaged mitochondria by triggering mitophagy [208], and, on the other hand, participates in the mutual regulation between pyroptosis and autophagy by regulating the ROS-AMPK-mTOR axis [209]. Study also demonstrated that the NLRP3 activation is related to the release of Cathepsin B (CTSB) induced by autophagy [210].

Finally, the link between pyroptosis and ferroptosis is through NRF2, which has effects on both inflammation inhibition and activation. NRF2 plays roles in ferroptosis as mentioned above, and it can also activate NLRP3 and AIM to induce pyroptosis. But, interestingly, NRF2 can also decrease the level of intracellular ROS to suppress NLRP3 activation, blocking pyroptosis [211, 212]. In general, most findings about the crosstalk of the 4 PCDs were obtained in the research of neurological diseases, immune diseases, or other types of cancer. These crosstalks may provide clues for exploring the links between various PCDs in GBM.

## Conclusions and Perspectives

In this review, we summarized the most latest researches on pyroptosis, ferroptosis, and autophagy in GBM. Pyroptosis in GBM is regulated by certain intracellular ncRNAs, and some drugs have been discovered to inhibit GBM growth by inducing pyroptosis. The expression of GSDM family members has potential to be used as biomarkers in clinical pathological diagnosis of GBM. Ferroptosis is associated with the elevated lipid peroxidation and iron levels. Ferroptosis engages with many pivotal signaling pathways in GBM, including those that govern oxidative stress, ER stress, cell proliferation, iron metabolism, lipid metabolism, and even autophagy. GBM has a higher iron metabolism level in response to abnormal proliferation, which is useful for exploring FINs to target ferroptosis. In the survival/death mechanism of GBM cell, autophagy is considered as a double-edged sword. However, we remain confident that there is an inevitable connection between autophagy and the tumorigenesis of GBM. There are various genetic alterations in molecules (IDH, EGFR, TP53, and AKT) involved in autophagy-associated GBM progression. Furthermore, P62, Beclin1, ATG5, and other molecules were found to have the impact on GBM progression. The unique hypoxic TME in GBM and the tolerance to clinical chemotherapeutic treatments were also linked to autophagy. Finally, determining how to felicitously use cytotoxic autophagy to kill GBM cells or inhibit protective autophagy, and ameliorating the autophagy-related drug tolerance caused by long-term administration of anti-glioma drugs, are still the two primary problems that remain to be solved.

## Supplementary Information

**Additional file 1.**

## Data Availability

Not applicable.
