# Supplementary figures and images for: Pyroptosis, ferroptosis, and autophagy cross-talk in glioblastoma opens up new avenues for glioblastoma treatment
Source: Cell Commun Signal. 2023 May 19;21:115. doi: 10.1186/s12964-023-01108-1 (PMC10199557; doi:10.1186/s12964-023-01108-1)

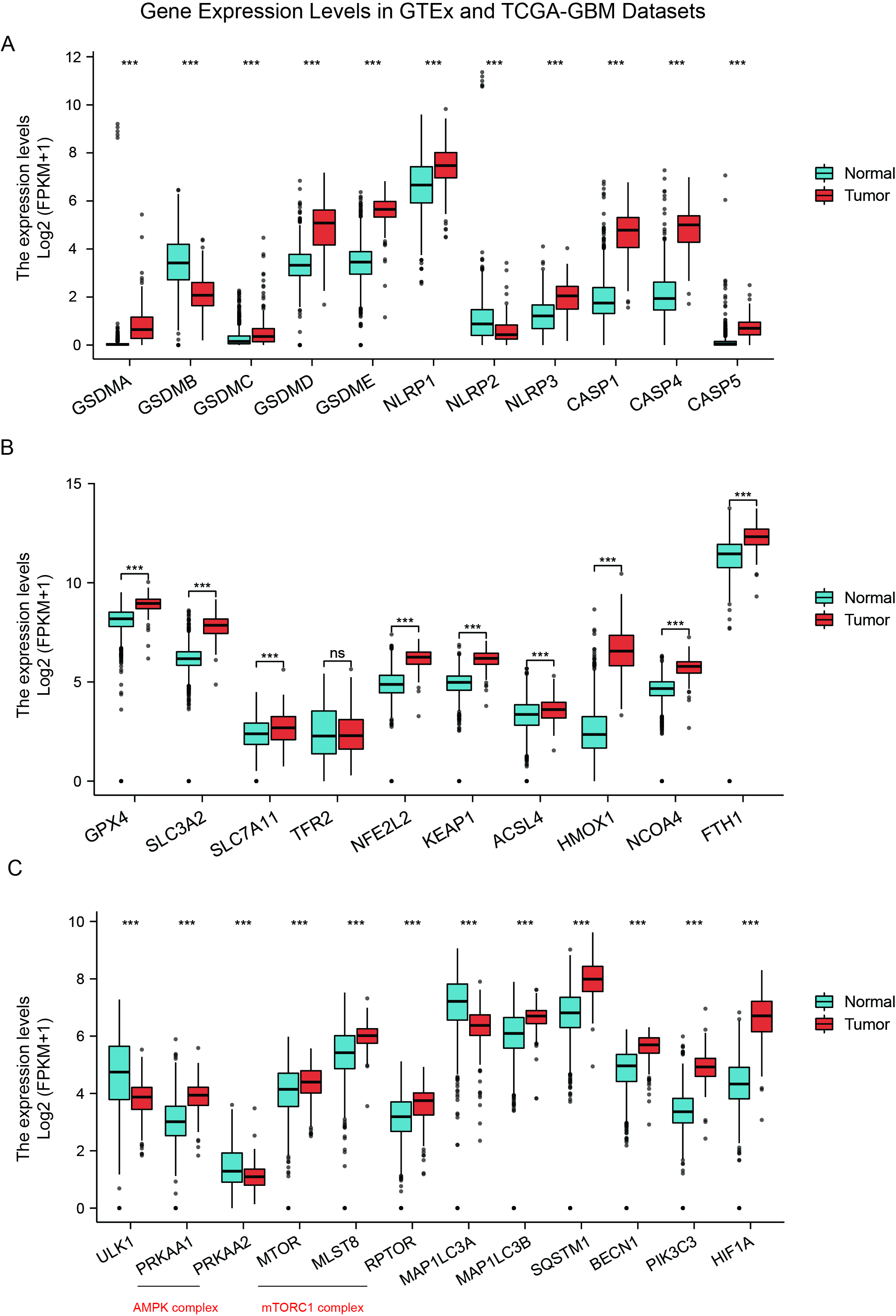

Supplement: Supplementary file 2 — Additional file 1. [file 12964_2023_1108_MOESM1_ESM.jpg]
